# CDK12-Mediated Phosphorylation of FOXA1 Promotes Prostate Cancer Progression via the MDM2–p53 Axis

**DOI:** 10.34133/research.0990

**Published:** 2025-11-10

**Authors:** Binyuan Yan, Mengjun Huang, Jinxiang Wang, Hanqi Lei, Tongyu Tong, Bin Xu, Fei Cao, Qiliang Teng, Jinsheng Huang, Yupeng Guan, Wenjun Mao, Haojie Huang, Jun Pang

**Affiliations:** ^1^ Department of Urology, Pelvic Floor Disorders Center, The Seventh Affiliated Hospital, Sun Yat-sen University, Shenzhen 518107, China.; ^2^ Department of Biochemistry and Molecular Biology, Mayo Clinic College of Medicine and Science, Rochester, MN 55905, USA.; ^3^ Department of Thoracic Surgery, The Affiliated Wuxi People’s Hospital of Nanjing Medical University, Wuxi People’s Hospital, Wuxi Medical Center, Nanjing Medical University, Wuxi 214023, Jiangsu, China.; ^4^ Department of Urology, The First Affiliated Hospital, Zhejiang University School of Medicine, Hangzhou 310003, China.; ^5^ Institute of Urologic Science and Technology, The First Affiliated Hospital, Zhejiang University School of Medicine, Hangzhou 310003, China.

## Abstract

Prostate cancer (PCa) progression is driven by intricate molecular mechanisms involving dysregulated signaling networks and posttranslational modifications of key regulatory proteins. In this study, we identify a novel oncogenic pathway wherein cyclin-dependent kinase 12 (CDK12) physically interacts with and phosphorylates forkhead box A1 (FOXA1) at serine 234 (S234). Phosphorylation at this residue markedly enhances FOXA1 transcriptional activity, leading to up-regulation of downstream targets including murine double minute 2 (MDM2), a critical negative regulator of the p53 tumor suppressor. Mechanistically, this CDK12–FOXA1–MDM2 axis destabilizes p53, attenuates apoptotic signaling, and promotes PCa cell survival and proliferation. Therapeutic targeting of CDK12 using the small-molecule inhibitor THZ531 or RNA interference effectively abrogates FOXA1 phosphorylation, restores p53 stability, reactivates apoptotic pathways, and suppresses tumor growth. Notably, the identification of S234 as a functional phosphorylation site in FOXA1 reveals a previously uncharacterized posttranslational regulatory mechanism in PCa biology. These findings establish the CDK12–FOXA1–MDM2 axis as a pivotal driver of PCa progression and underscore the therapeutic potential of targeting FOXA1 phosphorylation to restore tumor suppressor function and induce apoptosis in PCa. Our work provides a mechanistic framework for developing precision therapies aimed at disrupting this oncogenic cascade in PCa.

## Introduction

Prostate cancer (PCa) is the most frequently diagnosed malignancy in men worldwide [1], with androgen receptor (AR) signaling acting as a central driver of tumor growth and survival [2]. Although advancements have been made in AR pathway inhibitors, around 30% of patients still advance to metastatic stages like metastatic castration-resistant prostate cancer (mCRPC) and neuroendocrine prostate cancer, which pose considerable clinical difficulties [3]. Recent research has increasingly focused on AR coregulators to address therapeutic resistance to AR-targeted treatments. Among the key players driving these aggressive phenotypes, the transcription factor forkhead box A1 (FOXA1) has been identified as a critical regulator of AR reprogramming, enabling oncogenic transcriptional programs during cancer progression [4,5]. It seems that FOXA1 is a feasible breakthrough to overcome the treatment bottleneck, but the regulatory mechanism behind it has not been fully elucidated.

FOXA1, the third most commonly mutated gene in PCa, is crucial in managing transcriptional networks vital for the disease’s pathogenesis and progression [5–9]. Emerging evidence has revealed that functional alterations of FOXA1, driven by coding and noncoding mutations, significantly contribute to PCa progression [10–13]. Furthermore, the activity of FOXA1 is intricately regulated by cis-regulatory elements and posttranslational modifications, both of which represent promising avenues for therapeutic intervention [14–16]. Despite progress, the intricate mechanisms through which FOXA1 influences PCa progression are not fully understood, requiring further research to clarify its role and therapeutic potential.

Cyclin-dependent kinase 12 (CDK12) is a vital cyclin-dependent kinase, chiefly known for its function in gene transcription regulation during the cell cycle [17,18]. Beyond its canonical functions in transcriptional regulation, CDK12 has emerged as a significant driver in PCa [19]. Genetic alterations in CDK12 are strongly associated with tumor progression, metastasis, and resistance to therapy, highlighting its critical role in PCa pathogenesis [20]. Patients with CDK12 mutations often exhibit aggressive tumor phenotypes characterized by genomic instability and poor therapeutic outcomes, underscoring the kinase’s importance in maintaining genomic integrity and modulating cellular responses to treatment [21,22]. Intriguingly, CDK12 is increasingly recognized as a pleiotropic kinase, influencing various aspects of gene expression through its ability to phosphorylate substrates beyond the C-terminal domain (CTD) of RNAP II [23]. Exploration of the molecular mechanisms underlying CDK12’s role in PCa progression, including its interactions with protein complexes and potential non-CTD targets, could unveil novel therapeutic opportunities.

This study establishes FOXA1 as a genuine substrate of CDK12, identifying serine 234 (S234) as the phosphorylation site, which contributes to antiapoptotic effects and enhances PCa cell proliferation. Mechanistically, CDK12 phosphorylates the S234 residue of FOXA1, enhancing its chromatin binding ability and subsequently up-regulating the expression of downstream target genes, including MDM2. This process affects apoptotic pathways and alters the stability of the tumor suppressor p53 via the CDK12–FOXA1–MDM2 axis, promoting the progression of PCa in both in vitro and in vivo settings. The inhibition of CDK12, via the small-molecule THZ531 or RNA interference, markedly diminishes FOXA1 phosphorylation activity. This inhibition suppresses tumor growth by down-regulating antiapoptotic proteins and curbing the degradation of p53, thereby restoring apoptotic pathways. In summary, our study identified a novel phosphorylation site (S234) on FOXA1 and demonstrated that posttranslational modification at this site regulates apoptotic pathways and affects cell survival. These findings suggest a novel therapeutic approach targeting FOXA1 posttranslational modifications to hinder PCa progression, laying the groundwork for developing innovative treatments.

## Results

### Inhibition of CDK12 reduces FOXA1 response element activity

**Fig. 1.**
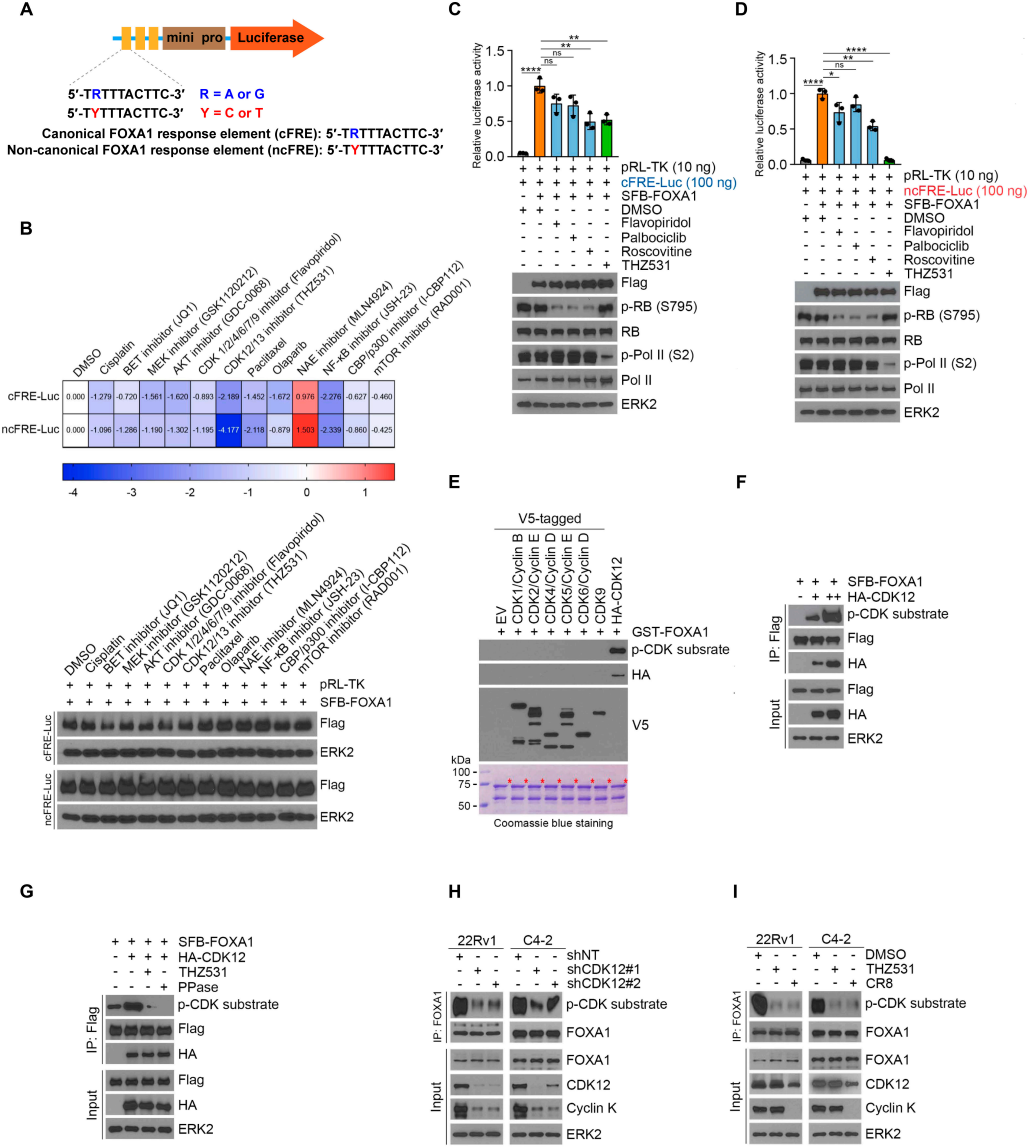
Inhibition of CDK12 reduces FOXA1 response element activity. (A) Schematic of FOXA1 luciferase reporter, depicting the modified response elements cloned in tandem upstream of a minimal promoter driving luciferase expression. (B) Heatmap depicting the sensitization scores (relative luciferase activity values for the indicated drug, log_10_ transformed) for a panel of inhibitors targeting potential oncogenic drivers (top), and immunoblot analysis of FOXA1 (Flag) levels as indicated. (C and D) FOXA1 luciferase reporter activity in response to treated with different CDK inhibitors. Data from 3 biological replicates; central line and error bars represent mean ± standard deviation. (E) Glutathione S-transferase (GST) pull-down assay using cells expressing V5-tagged CDK1, 2, 4, 5, 6, and 9 and HA-CDK12 and GST-tagged recombinant FOXA1 proteins. Asterisk, the expected proteins. (F) HEK293T cells were transfected with Flag-FOXA1 and increasing amounts of HA-CDK12, followed by Flag-IP and Western blotting analyses with the indicated antibodies. (G) HEK293T cells were transfected with Flag-FOXA1 and HA-CDK12, then treated with THZ531 or PPase, and followed by Flag-IP and Western blotting analyses with the indicated antibodies. (H) Western blot analysis of input and co-IP samples from C4-2 and 22Rv1 cells transfected with shNT or shCDK12 plasmids. (I) Western blot analysis of input and co-IP samples from C4-2 and 22Rv1 cells after treatment with 100 nM THZ531 or 100 nM CR8.

FOXA1 proteins are difficult to inhibit directly because of their complex mechanisms of action [24]. We created a FOXA1 reporter construct [10] (Fig. 1A) and designed inhibitors targeting key molecular pathways commonly implicated in cancer cell proliferation, survival, and apoptosis. Luciferase reporter assay results showed that NEDD8 activating enzyme inhibitor MLN4924 resulted in increased transcriptional activity, whereas THZ531, the inhibitor of CDK12/13, markedly impaired the activity (Fig. 1B). We then repeated the reporter assays by using the inhibitor of CDK1/2 (Flavopiridol), CDK4/6 (Palbociclib), CDK5 (Roscovitine), and CDK12/13 (THZ531). Consistently, THZ531, the CDK12/13 inhibitor, exhibited substantial inhibition of transcriptional activity, especially on the noncanonical sequence (Fig. 1C and D). The cross-link between CDK12 (which we previously reported to play a key role in PCa progression and enzalutamide resistance [25]) and FOXA1 attracted our interest. The consensus amino acid sequence for CDK substrates is (K/R)(S*)PX(K/R), where X represents any 1 of the 20 amino acids and S* denotes the phosphorylation site [26]. The pCDK substrate antibody serves as a crucial tool for identifying phosphorylation events mediated by CDKs, enabling precise detection of CDK activity and substrate phosphorylation in various biological contexts. Considering the kinase function of the CDK family [27], we next used the antibody to examine whether FOXA1 is a bona fide substrate of CDK12. We conducted an in vitro glutathione S-transferase (GST) pull-down assay to explore the kinase activity of the CDK family and discovered that FOXA1 is specifically phosphorylated by CDK12 (Fig. 1E). The phosphorylation level of FOXA1 rose as the CDK12 transfection dose increased (Fig. 1F). This trend was effectively reversed upon the introduction of THZ531 or a phosphatase (Fig. 1G). Both the small-molecule inhibitor THZ531 and RNA interference technology effectively reduced FOXA1 phosphorylation through functional inhibition of CDK12 (Fig. 1H and I). The same results were observed using Cyclin K degradator CR8, which implies a functional role for the CDK12–cyclin K complex in the phosphorylation of FOXA1(Fig. 1I). Together, these findings demonstrate that CDK12 affects the transcriptional activity of FOXA1 by phosphorylating FOXA1.

### The interaction between CDK12 and FOXA1

**Fig. 2.**
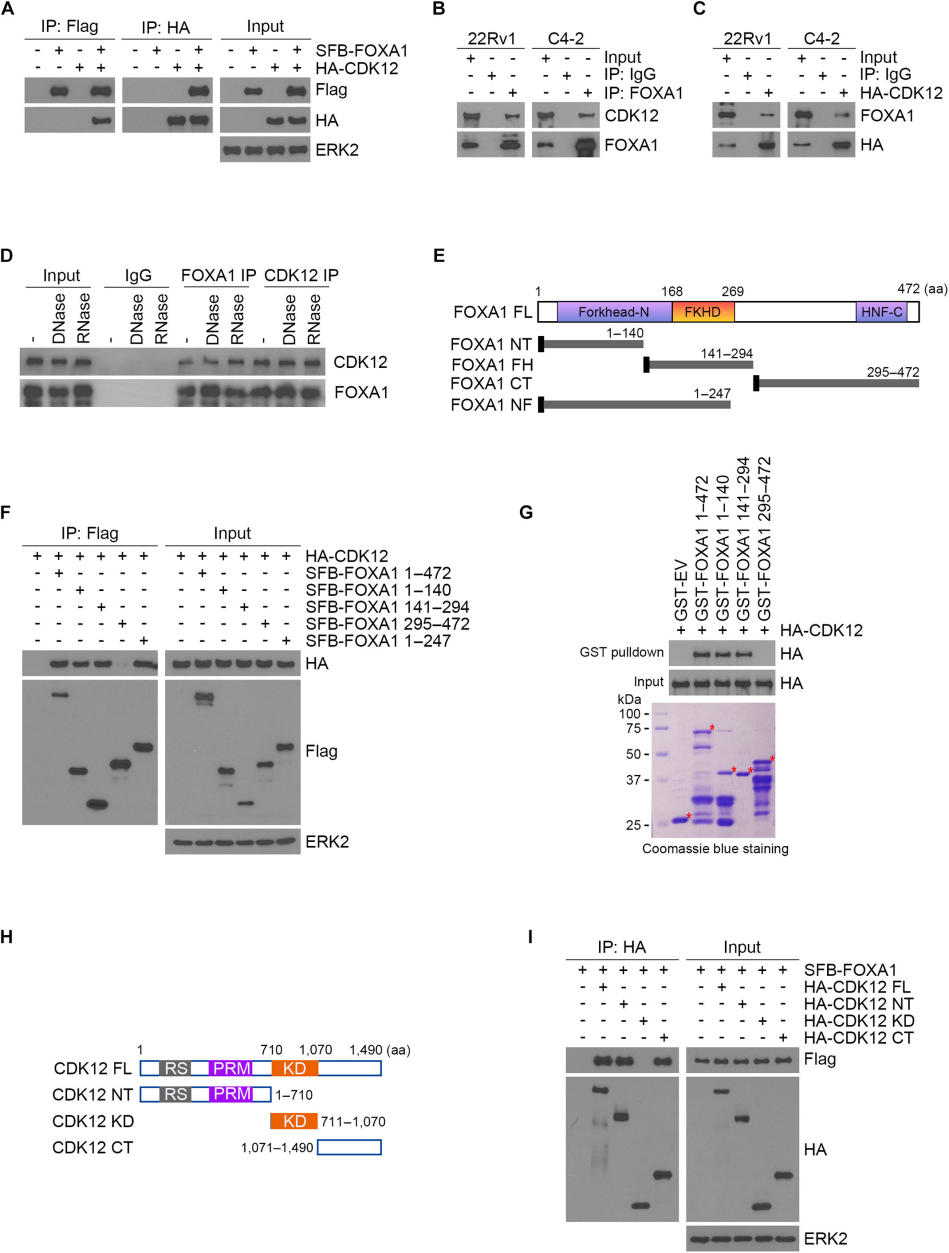
The interaction between CDK12 and FOXA1. (A) Western blot analysis of input and co-IP samples from HEK293T cells transfected with HA-CDK12 and Flag-FOXA1 plasmids. (B and C) Co-IP showing the endogenous CDK12 and FOXA1 interaction in C4-2 and 22Rv1 cells. (D) Western blot analysis of input and co-IP samples from C4-2. The co-IP samples were previously treated with DNase or RNase. (E) Schematic diagram of indicated FOXA1 truncation constructs. (F) 293T cells were transfected with HA-CDK12 and a series of FOXA1 truncation mutants, followed by Flag-IP and Western blotting analyses with the indicated antibodies. (G) GST pull-down assay using cells expressing HA-CDK12 and GST-tagged recombinant proteins. Asterisk, the expected proteins. (H) Schematic diagram of indicated CDK12 truncation constructs. (I) 293T cells were transfected with Flag-FOXA1 and a series of CDK12 truncation mutants, followed by HA-IP and Western blotting analyses with the indicated antibodies.

To further explore the potential interaction between CDK12 and FOXA1 proteins, we conducted reciprocal co-immunoprecipitation (co-IP) assays. The assays revealed a strong interaction between ectopically expressed CDK12 and FOXA1 in 293T cells, as well as between endogenous proteins in PCa cells (Fig. 2A to D). This interaction occurred independently of RNA or DNA (Fig. 2D). We generated various FLAG-tagged FOXA1 truncation mutants to pinpoint the essential domains for its interaction with CDK12 (Fig. 2E). Co-IP analysis revealed that full-length FOXA1 and its truncation variants encompassing residues 1–140, 141–294, and 1–247 maintained interaction with CDK12, while the 295–472 fragment lacked this ability (Fig. 2F). To confirm this direct interaction, GST pull-down assays using recombinant GST-FOXA1 truncation mutants and HA-tagged CDK12 proteins was performed again. The results showed that only the 295–472 fragment of FOXA1 failed to bind CDK12 (Fig. 2G). We created 3 HA-tagged CDK12 truncation mutants (Fig. 2H), and co-IP experiments indicated that the kinase domain of CDK12 is not essential for its interaction with FOXA1 (Fig. 2I). Collectively, these findings demonstrate a direct protein–protein interaction between FOXA1 and CDK12, with the 1–247 fragment of FOXA1 being critical for their binding.

### CDK12 directly phosphorylates FOXA1at S234

**Fig. 3.**
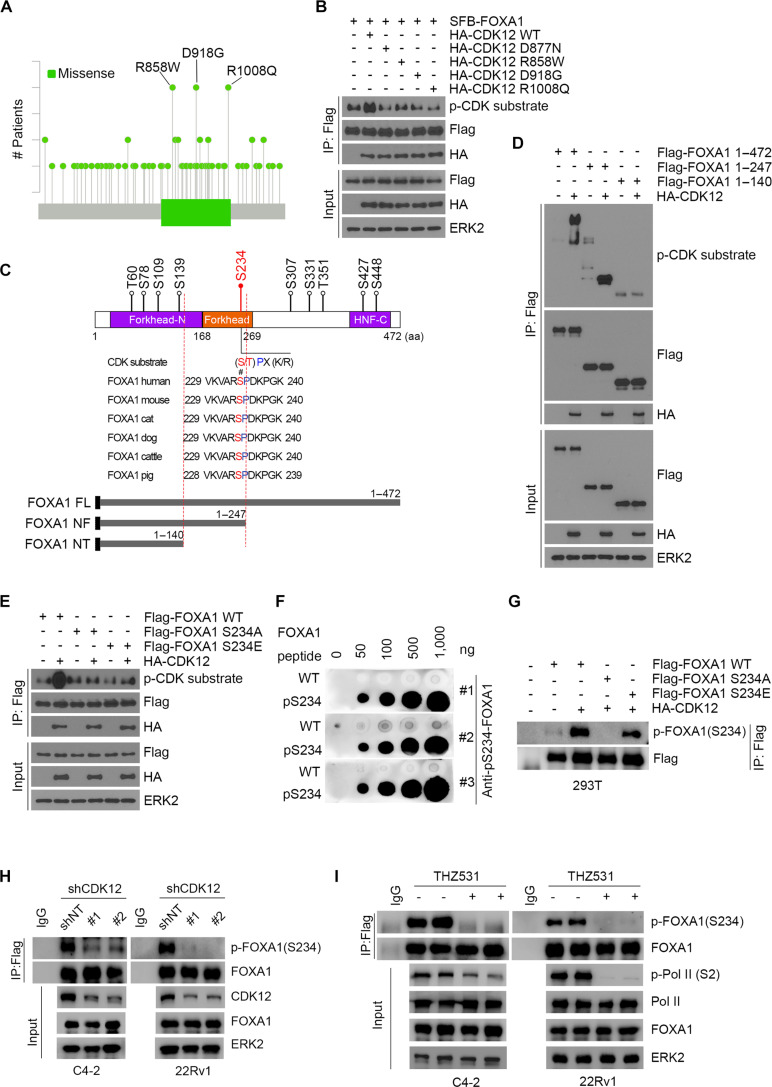
CDK12 directly phosphorylates FOXA1 at S234. (A) Spectrum of CDK12 gene mutations in prostate cancers in the cBioPortal database. (B) 293T cells were transfected with Flag-FOXA1 and a series of kinase-dead CDK12 mutants with different site mutations, followed by Flag-IP and Western blotting analyses with the indicated antibodies. (C) Putative FOXA1 phosphorylation sites based on reported proteomics data (PhosphoSitePlus) that are conserved across different species. Conserved serine and threonine residues are marked in red. (D) 293T cells were transfected with HA-CDK12 and indicated FOXA1 truncation plasmids, followed by Flag-IP and Western blotting analyses with the indicated antibodies. (E) 293T cells were transfected with HA-CDK12 and FOXA1 wild-type (WT), nonphosphorylatable (S234A), and phosphomimetic (S234E) mutants, followed by Flag-IP and Western blotting analyses with the indicated antibodies. (F) IB analysis of dot plot assay with indicated antibodies. (G) 293T cells were transfected with HA-CDK12 and Flag-FOXA1 WT, S234A, and S234E, followed by Flag-IP and Western blotting analyses with the anti-pFOXA1 S234 antibody. (H and I) IB analysis of input and IP products derived from PCa cells infected with CDK12 shRNAs (H) or treated with 100 μM THZ531 (I).

Given that FOXA1 can be phosphorylated by CDK12 as a bona fide substrate, we reasoned that CDK12 regulates the phosphorylation of FOXA1 in a kinase-dependent manner. To investigate this further, we conducted an analysis of somatic gene mutations in PCa samples using the cBioPortal platform. Our findings revealed a cluster of mutations in the kinase domain of CDK12, which includes 3 common mutations: R858W, D918G, and R1008Q (Fig. 3A). In a manner similar to the D877N mutation [28], which is recognized as a kinase-dead mutant of CDK12, the expression of these PCa-associated mutants consistently resulted in the loss of CDK12’s ability to phosphorylate FOXA1 (Fig. 3B).

To identify the phosphorylation sites on FOXA1 mediated by CDK12, we conducted a comprehensive analysis of posttranslational modifications of FOXA1 using the PhosphoSitePlus database (https://www.phosphosite.org/homeAction.action). The analysis revealed 5 potential phosphorylation sites within the CDK12-interacting domain of FOXA1 (AA1–247). Of these, 4 sites were situated within the AA1–140 segment, while the fifth site, S234, was located within the Forkhead domain of FOXA1 (Fig. 3C). We assessed the functional significance of these sites by co-transfecting 293T cells with CDK12 and 3 FOXA1 truncation mutants (AA1–472, AA1–247, and AA1–140) that interact with CDK12. Co-IP experiments revealed that the FOXA1 AA1–140 fragment was not phosphorylated by CDK12 (Fig. 3D). This finding suggests that S234, located within the Forkhead domain of FOXA1, is the primary phosphorylation site mediated by CDK12. Furthermore, S234 is highly conserved across vertebrates and aligns well with the S/TPXXS/T consensus motif characteristic of CDK substrates (Fig. 3C). We then generated phosphorylation site-specific mutants (S234A, a nonphosphorylatable mutant, and S234E, a phosphomimetic acid mutant) and conducted kinase assays. Compared to wild-type (WT) FOXA1, S234A mutation significantly reduced overall FOXA1 phosphorylation levels, indicating that S234 is a major phosphorylation site targeted by CDK12 (Fig. 3E). We also generated antibodies targeting FOXA1 phosphorylated at S234 (pS234-FOXA1) (Fig. 3F). Western blotting analyses using the anti-pS234-FOXA1 antibody confirmed that S234 is indeed phosphorylated by CDK12 (Fig. 3G to I). We further investigated whether CDK12-mediated phosphorylation of FOXA1 at S234 influences its nuclear–cytoplasmic shuttling. Nuclear–cytoplasmic fractionation assays combined with immunofluorescence analysis revealed that phosphorylation at the S234 residue does not alter the subcellular localization of FOXA1, indicating that this posttranslational modification does not regulate its nuclear import or cytoplasmic retention (Fig. S1).

### Phosphorylation of FOXA1 at S234 facilitates its DNA binding affinity and transcriptional activity

**Fig. 4.**
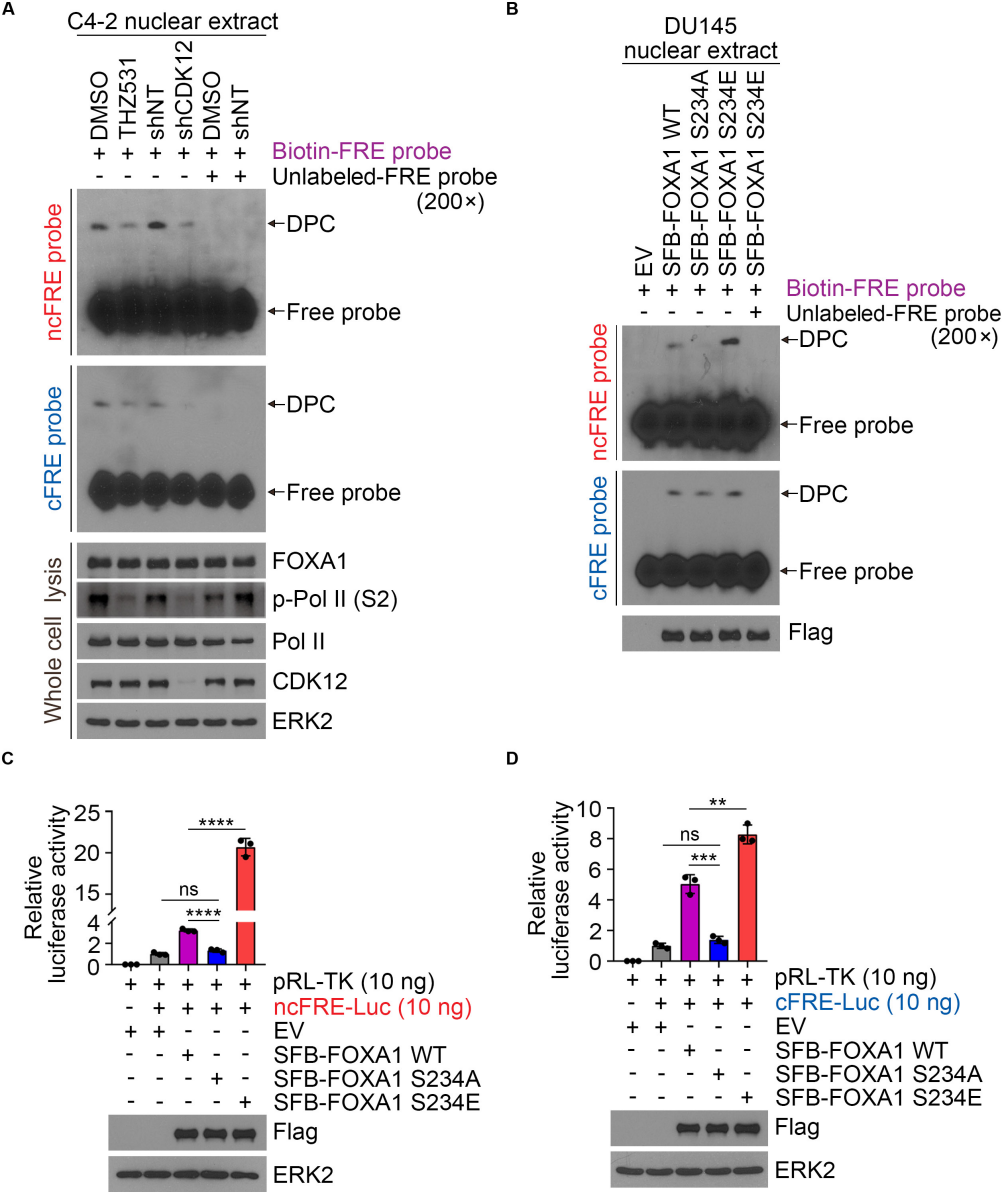
Phosphorylation of FOXA1 at S234 stables its chromatin binding and enhance its transcriptional activity. (A) C4-2 cells were treated with THZ531 or infected with shCDK12, then electrophoretic mobility shift assay (EMSA) was performed with nuclear extract. The upper band represents the DNA–protein complex (DPC), and the lower band indicates free probe. cFRE, canonical FOXA1 response element; ncFRE, noncanonical FOXA1 response element. (B) DU145 cells were transfected with FOXA1 WT, S234A, and S234E mutants, then EMSA was performed with nuclear extract. EV, as a negative control. (C and D) FOXA1 luciferase reporter activity in response to transfection with FOXA1 WT, S234A, and S234E mutants. EV, as a negative control.

Given that S234 situates in the Foxhead region of FOXA1, which is responsible for DNA recognition [29], we next assessed whether FOXA1 S234 phosphorylation influences its DNA binding affinity. We conducted an electrophoretic mobility shift assay (EMSA) on the nuclear components of PCa cells using a biotin-labeled DNA probe with a FOXA1 response element motif. The findings indicated that both pharmacological inhibition and gene knockdown of CDK12 significantly reduced FOXA1’s DNA binding compared to the control group (Fig. 4A). Consistently, EMSA results in DU145 expressing FOXA1 mutant constructs indicated that the phosphomimetic FOXA1 mutants (S234E) significantly enhanced FOXA1 binding to chromatin (Fig. 4B). We also repeated the luciferase reporter assays to assess the effect of FOXA1 S234 phosphorylation on its transcriptional activity and found that the nonphosphorylatable S234A mutation largely impaired its transcriptional activity on both the canonical (C/T) and noncanonical (A/G) sequence (Fig. 4C and D). Furthermore, molecular dynamics (MD) simulations demonstrated that FOXA1 harboring phosphorylation at S234 exhibits enhanced structural stability compared to its nonphosphorylated counterpart, suggesting that this posttranslational modification stabilizes the protein’s conformational state (Fig. S2). These results indicate that CDK12-mediated S234 phosphorylation of FOXA1 facilitates its DNA binding affinity and transcriptional activity.

### MDM2 is a downstream effector of the CDK12–FOXA1 axis regulating p53 protein stability

**Fig. 5.**
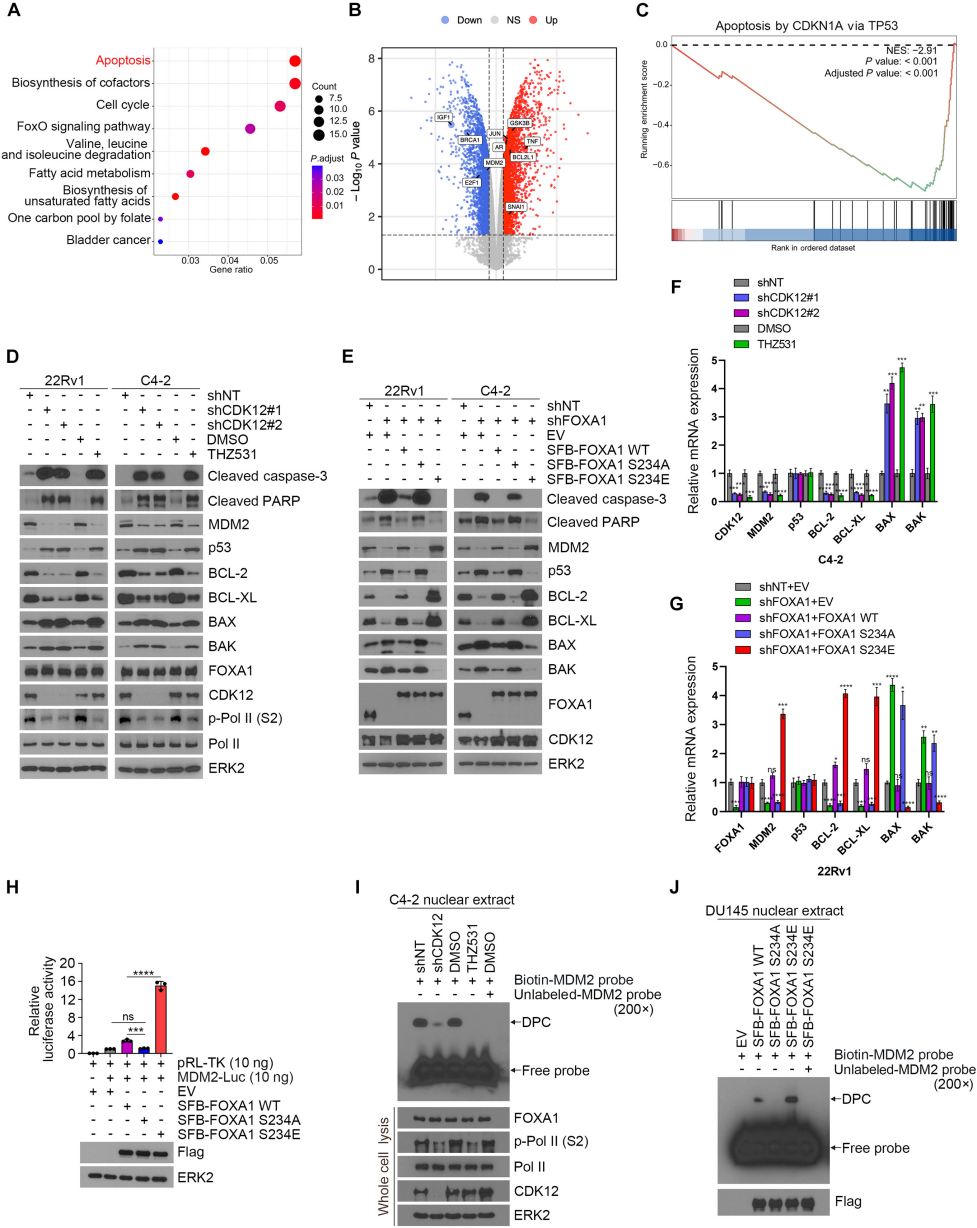
CDK12 phosphorylates FOXA1 to regulate apoptosis-related pathways and p53 protein stability. (A) The intersection of differentially expressed genes that were significantly reduced after THZ531 treatment and FOXA1 knockdown was used for KEGG pathway enrichment analysis. (B) A volcano plot of the differentially expressed genes after FOXA1 knockdown, and the apoptosis-related genes with the most significant expression differences were annotated. (C) Gene set enrichment analysis (GSEA) of apoptosis mediated by TP53 via Cdkn1a (p21) after knockdown of FOXA1. (D and F) mRNA and protein expression of apoptosis-related genes in C4-2 and 22Rv1 cells infected with CDK12 shRNAs or treated with 100 μM THZ531. (E and G) mRNA and protein expression of apoptosis-related genes in C4-2 and 22Rv1 cells that stably expresses the indicated plasmid. (H) Luciferase activity assay in response to transfection with FOXA1 WT, S234A, and S234E mutants. EV, as a negative control. (I) C4-2 cells were treated with THZ531 or infected with shCDK12, then EMSA was performed with nuclear extract using MDM2 promoter plasmid. The upper band represents the DPC, and the lower band indicates free probe. EV, as a negative control. (J) DU145 cells were transfected with FOXA1 WT, S234A, and S234E mutants, then EMSA was performed with nuclear extract using MDM2 promoter plasmid.

Given the established role of FOXA1 as a pioneer transcription factor in facilitating AR recruitment to chromatin, we further investigated whether CDK12-mediated phosphorylation at S234 of FOXA1 influences its pioneer function. Interestingly, our results demonstrated that neither the transcriptional nor translational levels of AR-regulated downstream targets KLK3 and NKX3-1 were altered by FOXA1 phosphorylation status (Fig. S3). This observation prompts further exploration of alternative signaling pathways activated by the CDK12–FOXA1 axis in PCa progression. We then performed a Venn diagram analysis to explore the downstream impact of CDK12-mediated phosphorylation of FOXA1, identifying numerous genes regulated by the CDK12–FOXA1 axis (Fig. S4A). Functional annotation via the Kyoto Encyclopedia of Genes and Genomes (KEGG) indicated significant enrichment of these genes in hallmark pathways related to apoptosis and the cell cycle (Fig. 5A). Given the previously uncharacterized relationship between FOXA1 and apoptosis, we further examined the transcriptional regulation of FOXA1 on genes involved in proliferation and apoptosis (Fig. 5B). The FOXA1 knockdown group exhibited a marked reduction in the characteristic signature of apoptosis (Fig. 5C). We then evaluated the transcriptional and protein expression levels of genes associated with apoptosis. Inhibiting CDK12 pharmacologically or through genetic knockdown significantly increased pro-apoptotic gene expression while decreasing antiapoptotic gene expression (Fig. 5D and F). Notably, overexpression of FOXA1 suppressed the expression of pro-apoptotic proteins and enhanced the expression of antiapoptotic proteins, with these effects being more pronounced in cells expressing the phosphorylation-mimetic FOXA1 S234E mutant (Fig. 5E and G). Furthermore, phosphorylation at S234 does not directly influence FOXA1’s transcriptional activity (Fig. 5E and G). These findings establish a functional link between the CDK12–FOXA1 axis and apoptosis, highlighting the role of FOXA1 phosphorylation in modulating apoptotic pathways.

Interestingly, we found that p53 protein levels were negatively regulated by CDK12–FOXA1, whereas its mRNA level did not change significantly, suggesting that CDK12-mediated FOXA1 phosphorylation affects the stability of p53 protein. We then intersected the significantly different expression genes after FOXA1 knockdown with apoptosis-related genes, and imported the result into cytoscape and used cytohubble to calculate core genes. Finally, we obtained the top 10 core genes (Fig. S4B). Using chromatin immunoprecipitation sequencing (ChIP-seq), we found that murine double minute 2 (MDM2), a key E3 ubiquitin ligase that catalyzes p53 ubiquitination, is directly transcriptionally regulated by FOXA1 (Fig. S4C and D). The luciferase assay and EMSA results reinforced that CDK12-mediated FOXA1 S234 phosphorylation facilitates the transcriptional output of MDM2 (Fig. 5H to J and Fig. S5). The correlation between CDK12 and MDM2 at the transcriptional and protein levels was further verified in the TIMER2.0 database (Fig. S6A) and tissue microarray (Fig. S6B and C). MDM2 acts as a key E3 ubiquitin ligase that catalyzes p53 ubiquitination through its evolutionarily conserved C-terminal really interesting new gene (RING) domain. This process involves both monoubiquitination, which promotes p53 polyubiquitination, ultimately leading to proteasome-mediated degradation of the tumor suppressor. Furthermore, we observed that CDK12 increased TP53 ubiquitination in a kinase-dependent manner and FOXA1 overexpression promoted TP53 degradation (Fig. S7). In summary, the findings suggest that CDK12-induced phosphorylation of FOXA1 enhances MDM2 transcription, promoting p53 ubiquitination and subsequent proteasomal degradation.

### Phosphorylated FOXA1 mediates CDK12’s role in promoting PCa growth

**Fig. 6.**
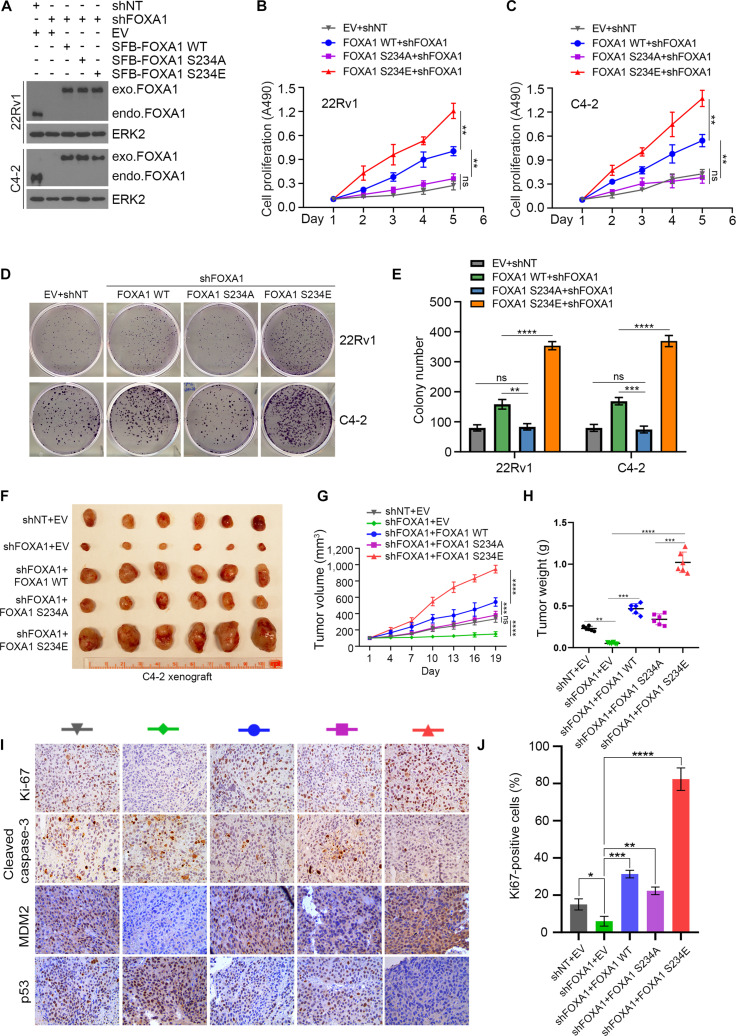
CDK12 inhibitor represses tumor growth partially by repressing FOXA1 activity. (A) Western blot analysis of FOXA1 after constructing PCa cell lines that stably express the indicated plasmid. (B to E) CCK8 and clonogenic growth assay of PCa cells that stably express the indicated plasmid. (F to H) Xenograft tumor growth curve and the collected tumors derived from C4-2 cells stably transfected with the specified plasmids. Tumor volume at various time points of treatment is measured using a vernier caliper. The quantitation data represent means ± SD, *n* = 6. (I and J) IHC analyses of Ki67, cleaved caspase 3, MDM2, and p53 protein expression in C4-2 cell-derived xenograft tissues upon treatment with THZ531.

To investigate whether phosphorylated FOXA1 functions as a cancer-promoting protein, we constructed PCa cell lines stably expressing FOXA1 WT, S234A, or S234E (Fig. 6A) and performed a series of functional experiments. The tumor-promoting effects of FOXA1 were validated through MTS assay and colony formation assay (Fig. 6B to E). Cell viability and EdU incorporation assays further confirmed the oncogenic role of FOXA1 phosphorylation at S234 (Fig. S8). Notably, simultaneous inhibition of CDK12 kinase activity and S234 phosphorylation synergistically suppressed the growth capacity of PCa cells, suggesting a critical regulatory axis in tumor progression (Fig. S8). We next determine the efficacy of phosphorylated FOXA1 by CDK12 in vivo. A nude mouse tumor xenograft model was established by inoculating stably transfected C4-2 cells subcutaneously. We found that FOXA1 knockdown significantly inhibited PCa growth in vivo. Overexpression of wild-type FOXA1 significantly promoted the malignant growth of tumors, and this phenomenon was exacerbated in tumors expressing FOXA1 S234E. Interestingly, the growth rate remained higher than that of control cells following rescue with the FOXA1 S234 mutant (Fig. 6F to H). Immunohistochemistry (IHC) staining confirmed the effects of the CDK12–FOXA1–MDM2 axis on p53 protein expression and cell proliferation (Fig. 6I and J). In summary, these data demonstrated that phosphorylated FOXA1 mediates the role of CDK12 in promoting PCa growth.

## Discussion

PCa is a highly heterogeneous disease shaped by diverse molecular aberrations. Notably, FOXA1 gene mutations have been observed in approximately 9% of primary PCa cases, with a higher incidence in CRPC [3,30,31]. In a cohort of Chinese patients, FOXA1 emerged as the most frequently mutated gene, with a mutation rate of 41% [32]. These genetic alterations have attracted considerable research interest due to their potential role in promoting tumor aggressiveness. Most missense and in-frame indel mutations in FOXA1, especially in the wing-2 domain, were identified as activating. Such mutations were associated with enhanced chromatin accessibility and increased transcriptional binding activity [32].

RNA and protein modifications are important mechanisms for PCa progression [33–35]. Recent studies have reported that posttranslational modification of FOXA1 affects its function. For example, phosphorylation of the FOXA1 S331 site has been implicated in the regulation of oxidative phosphorylation and cell proliferation in PCa cells, further highlighting its role in tumor growth and metabolic adaptation [36]. Lysine-specific demethylase 1 enhances FOXA1 chromatin binding by demethylating lysine 270, thereby reinforcing FOXA1’s role as a pioneer transcription factor for AR [15]. Notably, our study revealed that phosphorylation of FOXA1 at S234 by CDK12 significantly enhances its ability to promote PCa cell proliferation. This finding underscores the importance of posttranslational modifications in modulating FOXA1 function and highlights potential avenues for therapeutic intervention targeting these modifications.

Previous research has extensively documented the multifaceted roles of CDK12 in essential cellular processes, including DNA replication, transcription, splicing, and DNA damage repair [17]. These findings suggest that CDK12 not only plays a central role in transcriptional regulation during the cell cycle but also participates in broader cellular signaling processes. Building on this foundational understanding, our study identifies CDK12 as a novel modulator of FOXA1. CDK12 boosts FOXA1’s transcriptional activity through its kinase function, increasing FOXA1’s oncogenic potential in PCa cells. These results reveal a previously unrecognized role of CDK12 in regulating FOXA1 activity, shedding new light on the intricate molecular mechanisms driving PCa progression. Moreover, our study is the first to establish a direct link between CDK12, FOXA1, and apoptosis. We demonstrate that CDK12-mediated phosphorylation of FOXA1 enhances its transcriptional activation of antiapoptotic genes, tipping the apoptotic balance toward proliferation and survival. This finding underscores the role of the CDK12–FOXA1 axis in promoting tumor growth by modulating apoptotic pathways.

**Fig. 7.**
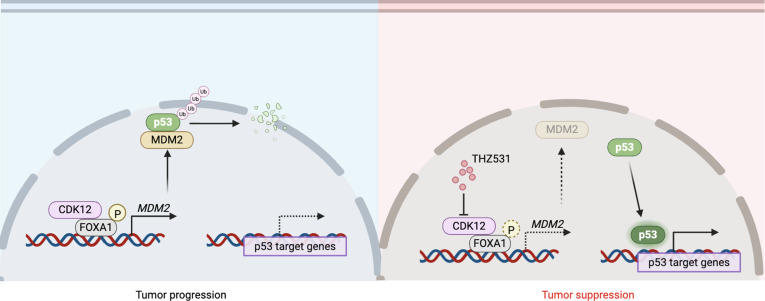
Illustration of the CDK12–FOXA1 axis in PCa progression. In PCa cells, CDK12 binds to and phosphorylates FOXA1 to promote the stability of FOXA1 and its ability to bind to chromatin, and promotes the expression of downstream target genes such as MDM2. MDM2 degrades P53 by ubiquitination to prevent it from exerting a tumor suppressor effect, thereby promoting the progression of PCa. By targeting and inhibiting CDK12 kinase activity, reducing FOXA1 phosphorylation, and down-regulating MDM2 to reduce its degradation of P53, the tumor suppressor effect is exerted, highlighting the potential role of CDK12 inhibitor for PCa therapies. The figure was generated with Biorender.com.

Although both p53 and FOXA1 are key factors implicated in cancer progression, their direct relationship remains poorly understood [37]. This study shows that FOXA1 influences p53 protein stability by regulating MDM2 expression, which, in turn, compromises p53’s tumor-suppressive functions. MDM2, a well-established negative regulator of p53, facilitates p53 ubiquitination and subsequent proteasomal degradation [38]. Our findings suggest that FOXA1 up-regulates MDM2 expression, thereby promoting the destabilization of p53 and hindering its tumor-suppressive activities. These insights highlight the potential of targeting the FOXA1–MDM2–p53 axis as a novel therapeutic strategy for cancers characterized by disrupted p53 activity (Fig. 7).

Recent studies have highlighted the intricate interplay between CDK12, AR, AR variants, and intronic polyadenylation [39]. Specifically, AR interacts with the promoter region of cyclin K to enhance its expression, which, in turn, activates the CDK12–cyclin K complex. This complex plays a critical role in suppressing intronic polyadenylation of the AR gene, thereby preventing the expression of AR variants. Consequently, inactivation of the CDK12–cyclin K complex leads to increased polyadenylation of the AR gene, resulting in elevated expression of AR variants. This, in turn, contributes to therapeutic resistance and progression to CRPC. Notably, 2 distinct types of CDK12 mutations have been identified in CDK12-mutant PCa, both of which promote intronic polyadenylation of AR and the expression of AR variants [40]. These findings underscore the importance of CDK12’s classical role in inhibiting intronic polyadenylation along the AR stabilization axis. In this study, we focused on elucidating the mechanism by which phosphorylation of FOXA1 at S234 regulates PCa cell proliferation and invasion. While our findings provide valuable insights into the functional role of FOXA1 S234 phosphorylation, we acknowledge that the broader implications of this modification on genome-wide transcriptional regulation and its potential influence on AR binding and associated transcriptional activities remain unexplored. These aspects are critical for a more comprehensive understanding of the role of FOXA1 S234 phosphorylation in PCa biology. Future research could incorporate genome-wide analyses to systematically investigate the regulatory effects of FOXA1 S234 phosphorylation on chromatin accessibility and gene expression. Additionally, exploring whether this phosphorylation event impacts the interaction between FOXA1 and AR, as well as its potential effects on PCa progression, could provide novel insights into this field. Such studies would not only enhance our understanding of the functional role of FOXA1 S234 phosphorylation in PCa but also potentially pave the way for the development of innovative therapeutic strategies.

In summary, our study uncovers a pivotal role of CDK12-mediated phosphorylation in enhancing FOXA1’s transcriptional activity and tumor-promoting functions, thereby driving PCa progression. Mechanistically, CDK12 phosphorylates FOXA1 at S234, leading to the transcriptional up-regulation of MDM2, a key negative regulator of the tumor suppressor p53. Notably, inhibition of CDK12 prevents p53 degradation, thereby restoring its tumor-suppressive activity and inducing apoptosis in PCa cells. The study underscores the pivotal role of the CDK12–FOXA1–MDM2–p53 axis in modulating cell survival and tumor progression. Our findings highlight the therapeutic potential of targeting this pathway, especially in PCa cases with abnormal CDK12–FOXA1 activity. By disrupting this oncogenic axis, it may be possible to restore apoptotic pathways and suppress tumor growth, offering a promising avenue for novel treatments for advanced PCa.

## Materials and Methods

### Cell lines and chemicals

C4-2 and 22Rv1 cells were maintained in RPMI 1640 medium (Corning), whereas HEK293T cells were grown in Dulbecco’s Modified Eagle Medium (DMEM) (Corning) supplemented with 10% fetal bovine serum, 100 mg/ml streptomycin, and 100 U/ml penicillin. Cells were maintained at 37 °C in a 5% CO₂ environment. The information regarding the chemicals used in this study is provided in Table S1.

### Stable cell-line generation

In summary, 293T cells were co-transfected with short hairpin RNA (shRNA) plasmids and lentiviral packaging plasmids (PSPAX2 and PMD2.G) using polyethyleneimine. The viral supernatant was collected, filtered, and used to infect target cells 48 h after transfection. Puromycin (1 μg/ml) was introduced after 48 h to select stable cell lines. The shRNA sequences are detailed in Table S2.

### DNA constructs

Various HA-CDK12 and Flag-FOXA1 deletion mutants were generated through polymerase chain reaction (PCR) amplification of CDK12 and FOXA1 cDNA, followed by cloning into pCMV-HA and pCMV-Flag vectors, respectively. Similarly, GST-FOXA1 deletion constructs were created by amplifying FOXA1 cDNA via PCR and subcloning into the pGEX-4T-1 expression vector. HA-CDK12 and V5-FOXA1 mutants were generated via site-directed mutagenesis using the KOD Plus Mutagenesis Kit (Toyobo) following the manufacturer’s instructions. The sequences of primers used for all cloning procedures are listed in Table S3.

### RNA isolation and qRT-PCR

RNA extraction was performed using TRIzol reagent, followed by reverse transcription with the GoScript Kit (Promega). Real-time PCR was performed using SYBR Green Master Mix (Bio-Rad). Primer sequences for quantitative PCR are detailed in Table S4.

### Antibodies and immunoblotting

For immunoblot analysis, cell lysates were prepared using a modified RIPA buffer with 1% protease inhibitor cocktail. Protein separation was performed via sodium dodecyl sulfate polyacrylamide gel electrophoresis (SDS-PAGE) (Bio-Rad), and the resolved proteins were transferred to polyvinylidene fluoride (PVDF) membranes (Thermo Fisher Scientific). The membranes were treated with 5% bovine serum albumin (BSA) at room temperature for 1 h, followed by an overnight incubation with primary antibodies at 4 °C. Information regarding the primary antibodies used is provided in Table S5.

### GST pull-down assay

GST-tagged fusion proteins were expressed in *Escherichia coli* BL21 (Thermo Fisher Scientific) and isolated using glutathione-Sepharose resin (GE Healthcare Life Sciences) according to the manufacturer’s protocol. For negative control purposes, an empty vector (pGEX-4T-1) was utilized, which produces GST without any additional fusion partners. The purified GST fusion proteins were incubated with total lysates from HEK293T cells transfected with the specified plasmids. Following a 2-h incubation, the resin was centrifuged to form a pellet and washed 5 times to eliminate nonspecifically bound components. Proteins bound to the resin were separated by SDS-PAGE and examined through Western blotting with the specified antibodies.

### Peptide synthesis and dot immunoblot assays

FOXA1-WT and FOXA1-pS234 peptides used for dot blot assays were synthesized by Abclonal. The sequences FOXA1-WT (KVARSPDKP-C) and FOXA1 pS234 [KVAR(pS)PDKP-C] were diluted to 1 mg/ml in phosphate-buffered saline (PBS) and spotted onto a nitrocellulose membrane in amounts of 0.05, 0.1, 0.5, and 1 μg. The membranes were then dried and blocked for immunoblot analysis.

### Xenograft experiments

Animal experiments received approval from the Mayo Clinic’s Institutional Animal Care and Use Committee (IACUC). Six-week-old male severe combined immunodeficiency mouse (SCID) mice were randomly assigned, and C4-2 cells with the specified plasmid were subcutaneously implanted in the mice’s right flank. Tumor size was measured every 3 days, and the volume was calculated using *a* * *b*^2^/2, where *b* is the shortest diameter of the tumor and *a* is the longest diameter perpendicular to *b*. The xenografts were collected for subsequent IHC analysis.

### Quantification and statistical analysis

Statistical analyses were conducted using GraphPad Prism 9. The data are presented as the mean ± standard error based on 3 independent experiments. An unpaired 2-tailed Student’s *t* test was used to analyze group differences, with statistical significance set at *P* < 0.05.

## Ethical Approval

The animal studies were conducted with approval from the IACUC at the Mayo Clinic.

## Data Availability

Data will be made available on request.
