# Supplementary material for: CDK12-Mediated Phosphorylation of FOXA1 Promotes Prostate Cancer Progression via the MDM2–p53 Axis
Source: Research (Wash D C). 2025 Nov 10;8:0990. doi: 10.34133/research.0990 (PMC12696697; doi:10.34133/research.0990)
Supplement: Supplementary 1 — Figs. S1 to S8 Tables S1 to S6 [file research.0990.f1.zip › Supplementary Figures.docx]

Supplementary Figures


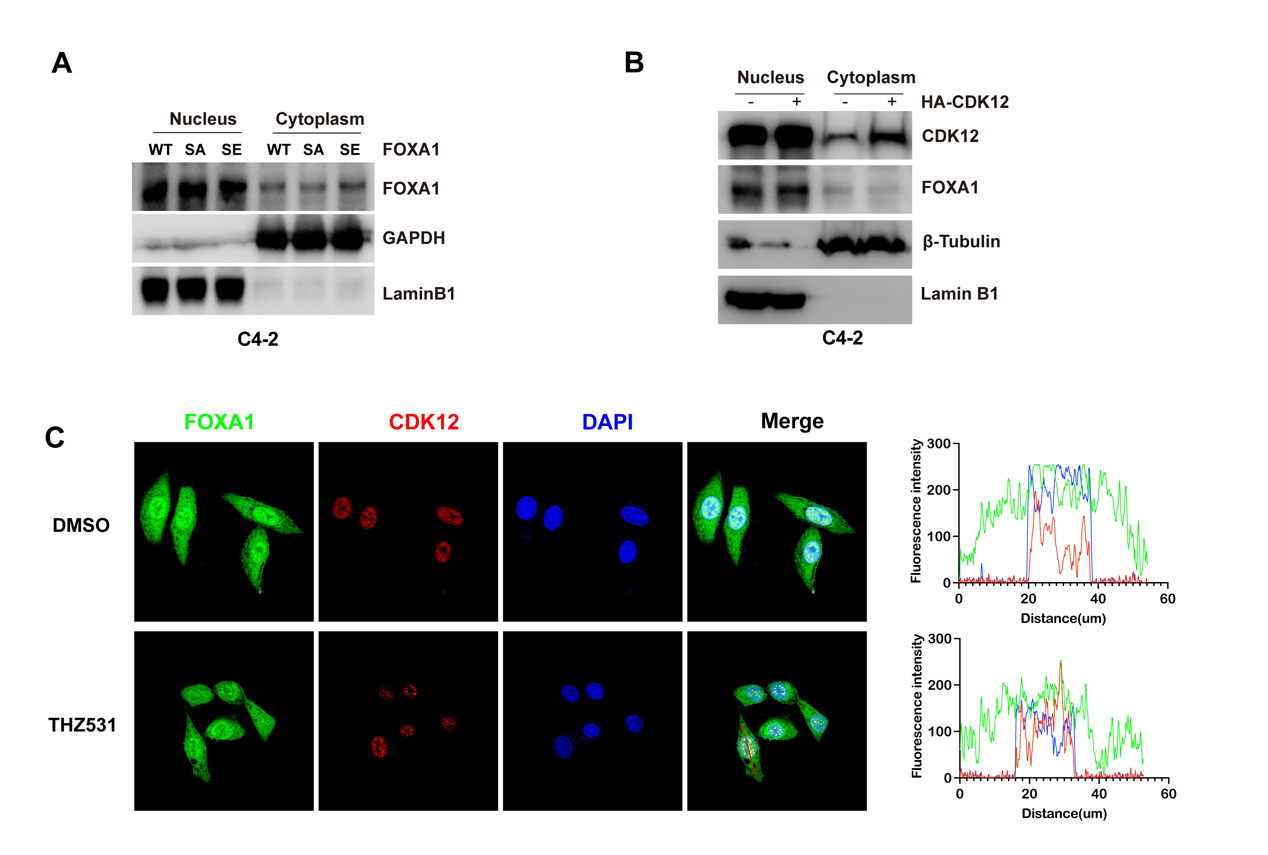


Figure S1. ​Phosphorylation of FOXA1 by CDK12 does not affect the nucleocytoplasmic shuttling of FOXA1.

(**A)** ​FOXA1 WT, S234A(SA), and S234E(SE) were reintroduced in shFOXA1 C4-2 cells, and the distribution of FOXA1 was detected by western blotting after nuclear and cytoplasmic separation. ​​​(**B)** ​​​HA-CDK12 was overexpressed in C4-2, and the distribution of FOXA1 was detected by western blotting after nuclear and cytoplasmic separation. (**C) A**fter C4-2 cells were treated with THZ531 (100 nM) for 12 h, the cellular localization of FOXA1 and CDK12 was analyzed by immunofluorescence.


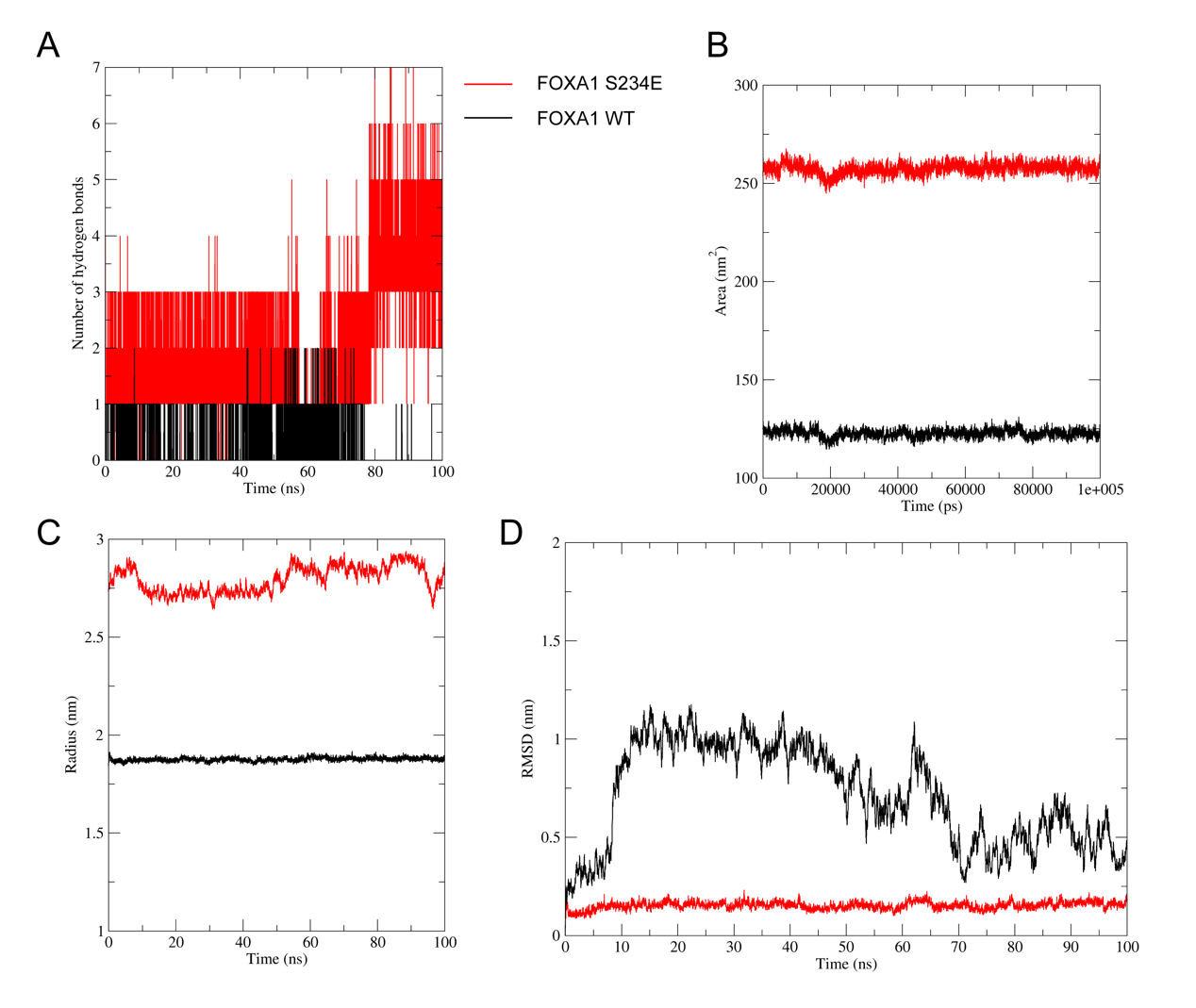


Figure S2. Molecular dynamics simulation analysis.

(**A)** ​Number of hydrogen bonds. ​​​(**B)** ​​​Time evolution of solvent accessible surface area (SASA) during molecular dynamics simulations. (**C)** Graph of gyration radius changing with time. (**D)** Root mean square deviation (RMSD) evolution over time during molecular dynamics simulations. The red curve represents the RMSD of FOXA1 phosphorylated at S234, which remained below 0.2 nm throughout the simulation, indicating a relatively stable structure of this molecule or portion of the molecule.


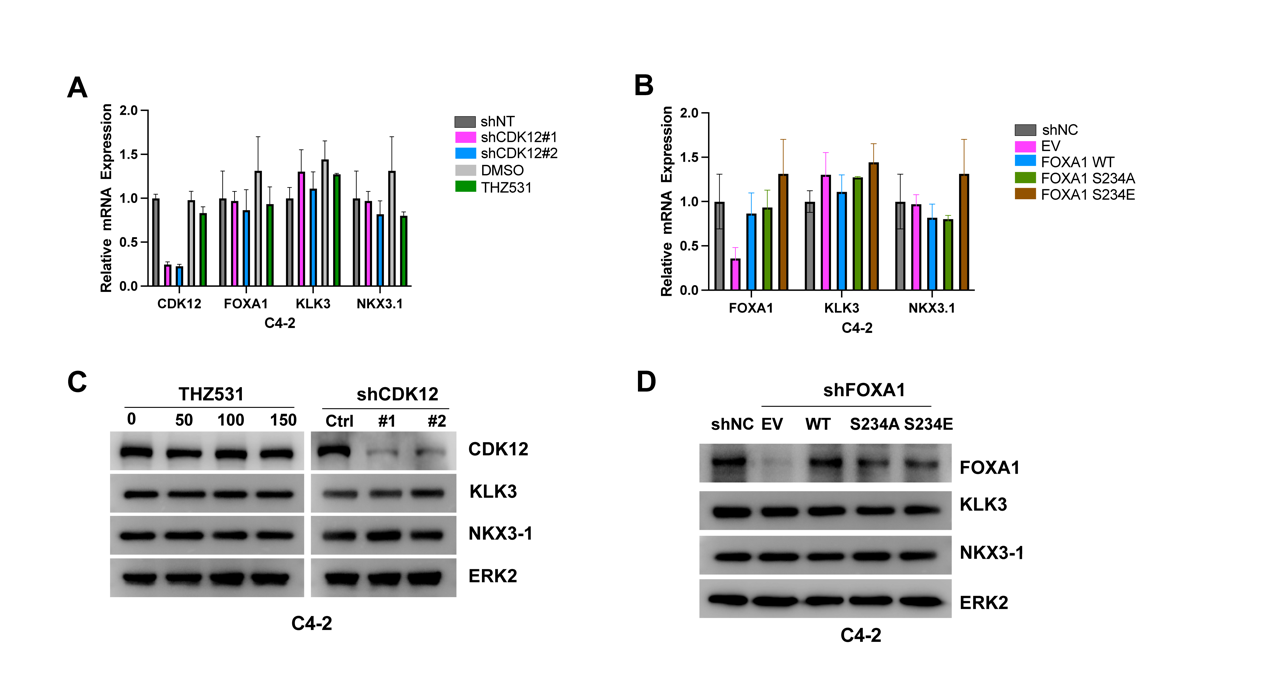


Figure S3. CDK12 phosphorylation of FOXA1 does not affect the transcriptional regulation of androgen receptor.

(**A)** ​mRNA levels of each gene after gene knockdown or pharmacological inhibition of CDK12. ​​​(**B)** mRNA levels of each gene after reintroducing FOXA1 WT, S234A(SA), and S234E(SE) in shFOXA1 C4-2 cells. ​​​(**C)** CDK12, KLK3, NKX3-1 was detected by western blotting after gene knockdown or pharmacological inhibition of CDK12. (**D)** FOXA1, KLK3, NKX3-1 was detected by western blotting after reintroducing FOXA1 WT, S234A(SA), and S234E(SE) in shFOXA1 C4-2 cells.


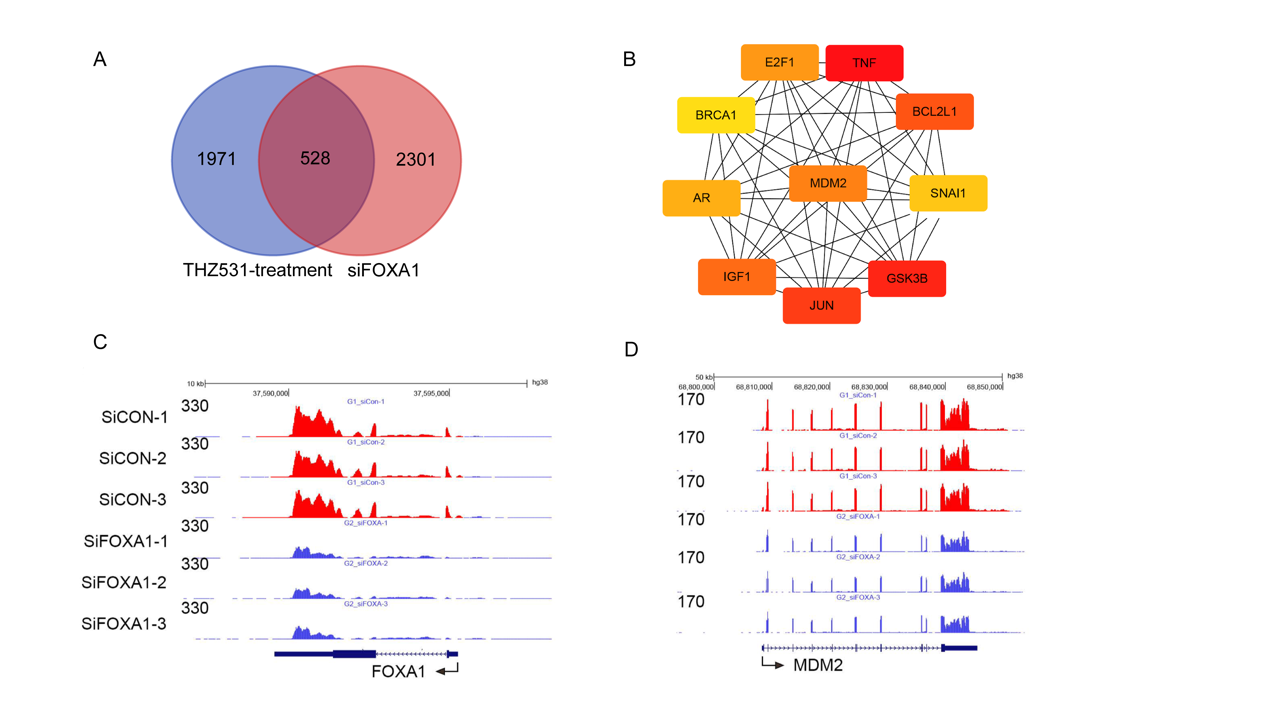


Figure S4. ​CDK12 phosphorylates FOXA1 to regulate apoptosis-related pathways.

​​(**A)** ​Venn diagram of the intersection of differentially expressed genes that were significantly reduced after THZ531 treatment and FOXA1 knockdown treatment. ​​​(**B)** ​​​The intersection genes from (A) were imported into Cytoscape and Cytohubble to calculate the core genes which was used for protein-protein interactions. (**C,D)** ​Chromatin immunoprecipitation sequencing (ChIP-seq) results of FOXA1 (C) and MDM2 (D).

​


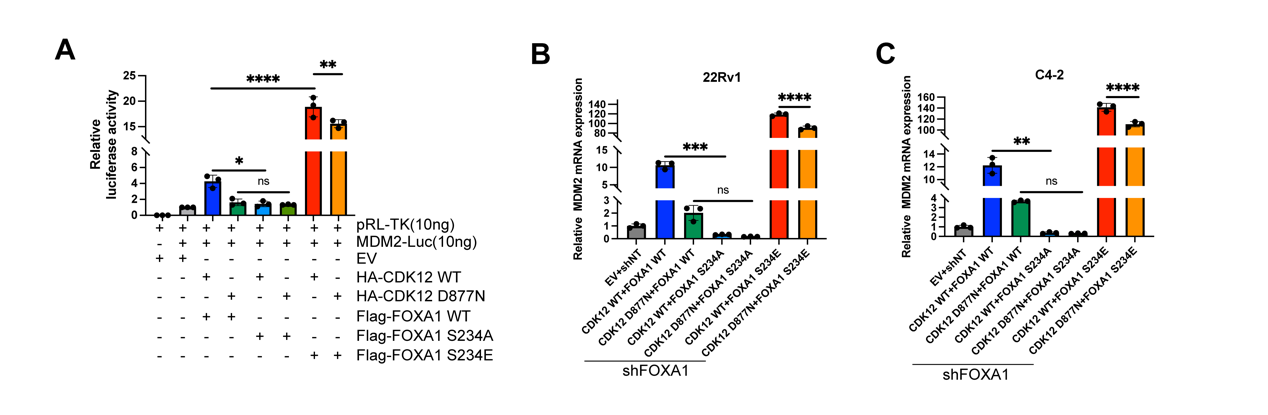


Figure S5. ​CDK12 activity affects the transcriptional activity of FOXA1 on MDM2.

(**A)** ​Luciferase activity assay in response to transfected with CDK12 WT, D877N, and FOXA1 WT, S234A, S234E mutants. EV, as a negative control. ​​​(**B,C)** MDM2 transcription levels were detected by qPCR after FOXA1 WT, S234A, and S234E mutants were reintroduced in FOXA1 knockdown PCa cells and CDK12 WT and D877N inactive mutants were overexpressed.​​​ ​


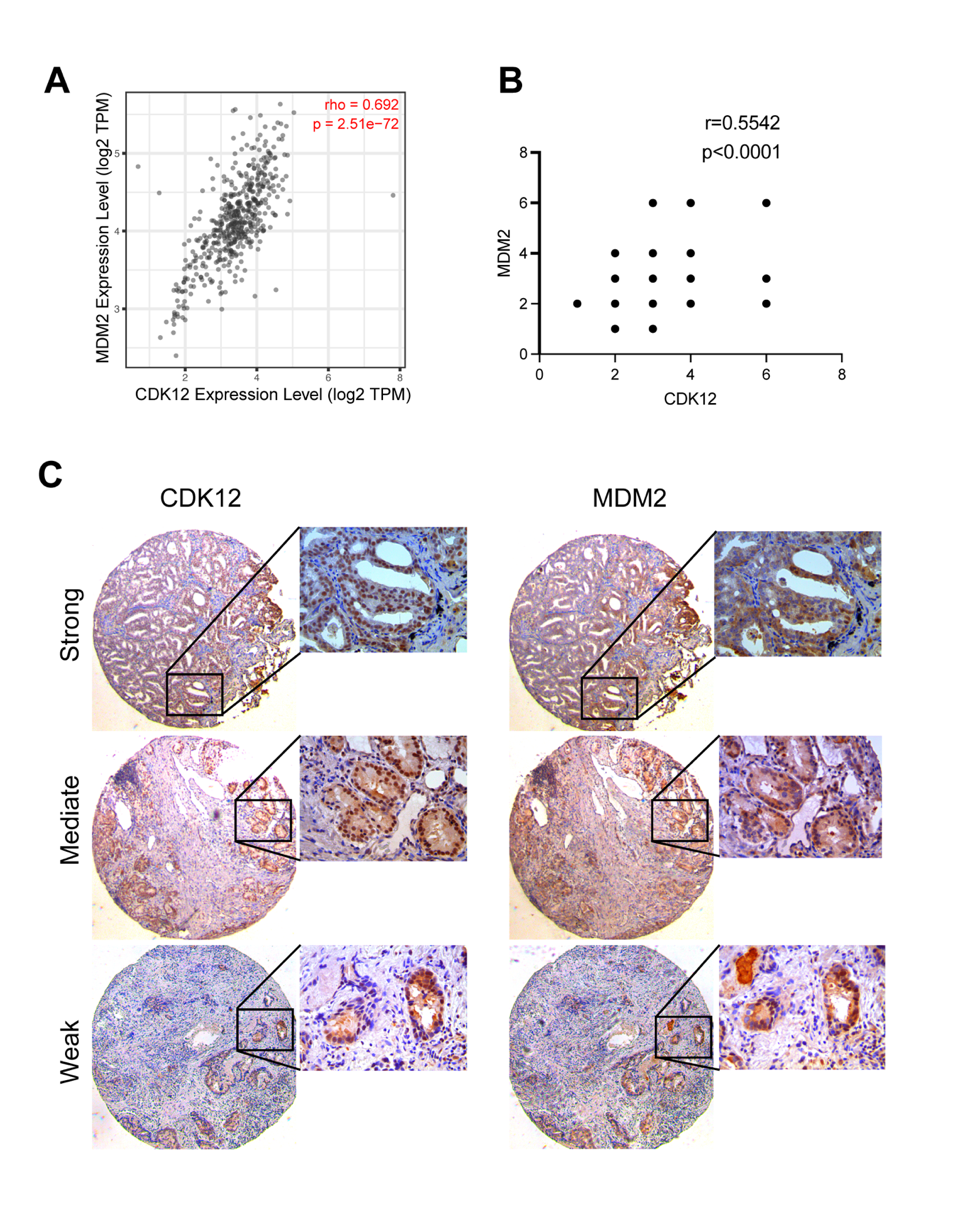


Figure S6. ​Correlation between CDK12 and MDM2 expression

​​​(**A)** ​Correlation between CDK12 and MDM2 ​mRNA expression in the TMER2.0 database. (​**B)** ​​Correlation between CDK12 and MDM2 protein expression. ​(**C)** ​Representative immunohistochemistry images show the correlation between different expression levels of CDK12 and MDM2 proteins.


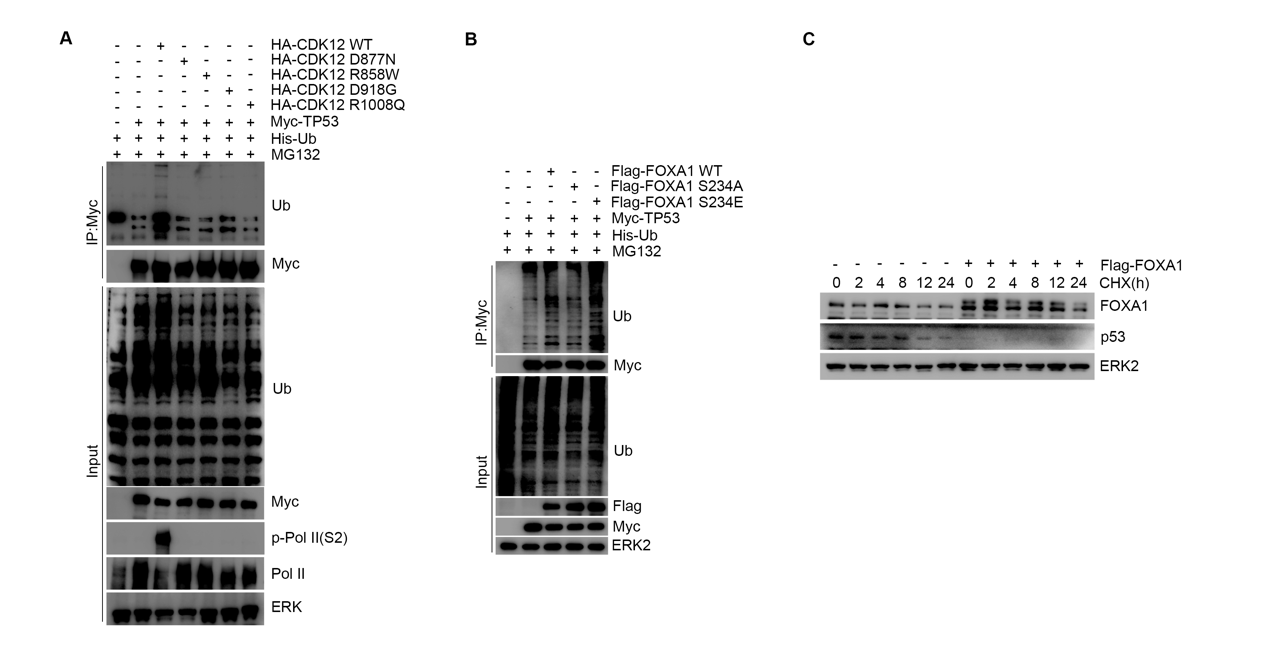


Figure S7. ​CDK12-mediated FOXA1 phosphorylation facilitates the ubiquitination of p53 protein, leading to the degradation of p53 by the proteasome.

(A,B) 293T cells were cotransfected with indicated plasmids and the polyubiquitylated TP53(Myc) protein was examined by western blot. (C) Cycloheximide pulse-chase assay was performed in 22Rv1 cells over-expressing FOXA1.


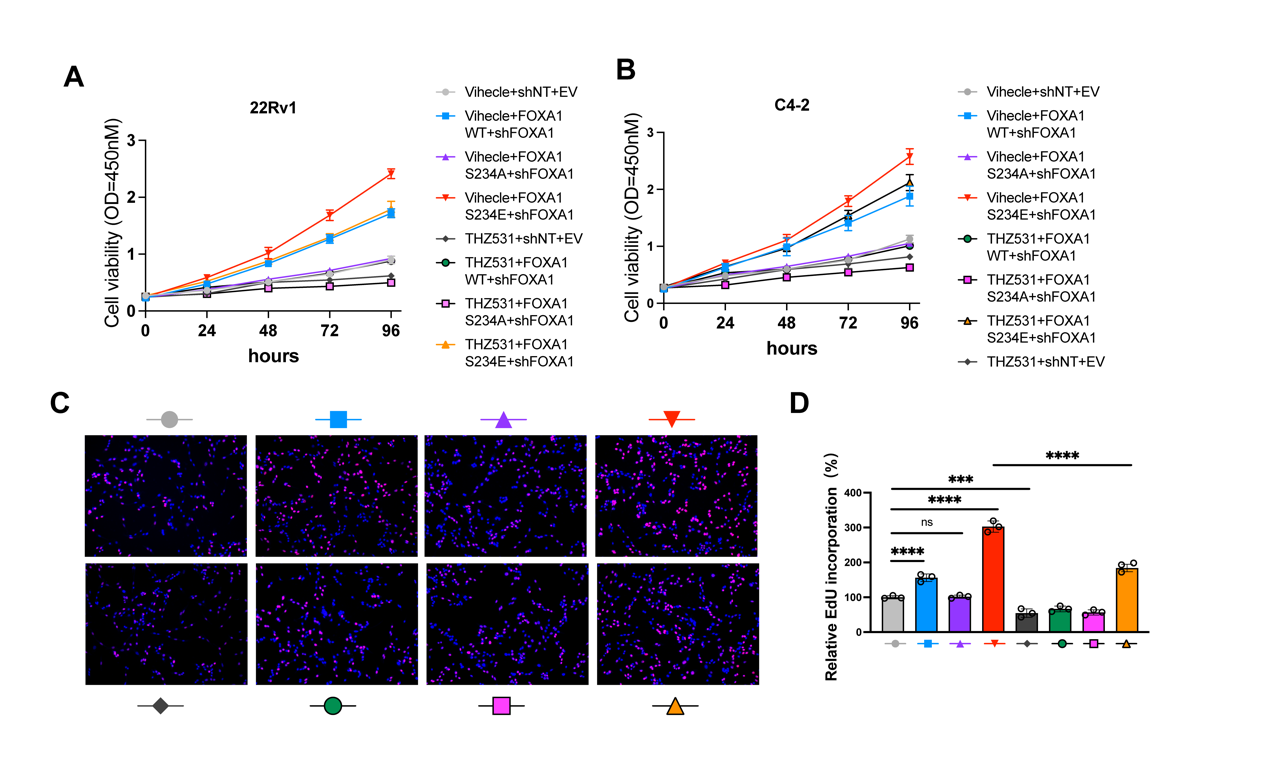


Figure S8. ​THZ531 pharmacologically inhibits CDK12 activity and weakens the viability and proliferation of PCa cells.

(A-C) Cell viability assay(A,B) and EdU assay(C) after FOXA1 WT, S234A, and S234E mutants were reintroduced in FOXA1 knockdown PCa cells and treated with THZ531 (100 nM) before cell viability assay.
