# Supplementary material for: CDK12-Mediated Phosphorylation of FOXA1 Promotes Prostate Cancer Progression via the MDM2–p53 Axis
Source: Research (Wash D C). 2025 Nov 10;8:0990. doi: 10.34133/research.0990 (PMC12696697; doi:10.34133/research.0990)
Supplement: Supplementary 1 — Figs. S1 to S8 Tables S1 to S6 [file research.0990.f1.zip › Supplementary Tables.docx]

**Supplementary Table 1. Information of chemicals**

| **Chemicals** | **Vendors** | **Cat#** |
| --- | --- | --- |
| THZ531 | TargetMol | T4293 |
| CR8 | Cayman | 14006 |
| Cisplain | MCE | HY-17394 |
| JQ1 | MCE | HY-13030 |
| GSK1120212 | MCE | HY-10999 |
| GDC-0068 | MCE | HY-15186 |
| Flavopiridol | Cayman | 10009197 |
| Paclitaxel | MCE | HY-B0015 |
| Olaparib | MCE | HY-10162 |
| MLN4924 | Cell Signaling Technology | 85923 |
| JSH-23 | Sigma | 481408 |
| I-CBP112 | Cayman | 14468 |
| RAD001 | MCE | HY-10218 |
| Roscovitine | Cayman | 10009569 |

**Supplementary Table 2. Sequences of shRNAs.**

| **Plasmids** | **Sequences** |
| --- | --- |
| shNS | CCGGCAACAAGATGAAGAGCACCAACTCGAGTTGGTGCTCTTCATCTTGTTGTTTTT |
| shCDK12#1 | CCGGGCACTGAAAGAGGAGATTGTTCTCGAGAACAATCTCCTCTTTCAGTGCTTTTT |
| shCDK12#2 | CCGGGCGTTCCCGGGACTTACTAAACTCGAGTTTAGTAAGTCCCGGGAACGCTTTTTG |
| shFOXA1#1 | GCGTACTACCAAGGTGTGTATctcgagATACACACCTTGGTAGTACGCttttt |

**Supplementary Table 3. Primers used for plasmid construction**

| **Genes** | **Primers** | **Sequences** |
| --- | --- | --- |
| HA-CDK12 FL | Forward | cggtcgaccATGCCCAATTCAGAGAGACATGGG |
|  | Reverse | cagcggccgcttaGTAAGGAACTCCTCTCCCTCTTCC |
| V5-FOXA1 | Forward | CTTATGTTAGGAACTGTGAAGATGGAAGGG |
|  | Reverse | TCCCTAGGAAGTGTTTAGGACGG |
| Flag-FOXA1 S234A | Forward | gtggcacgcGCcccg |
|  | Reverse | cgggGCgcgtgccac |
| Flag-FOXA1 S234E | Forward | gtggcacgcGAGccggaca |
|  | Reverse | tgtccggCTCgcgtgccac |
| HA-CDK12 NT | Forward | cggtcgaccATGCCCAATTCAGAGAGACATGGG |
|  | Reverse | cagcggccgcttaTCCATAACGAGGACAACAAATTTTT |
| HA-CDK12 KD | Forward | cggtcgaccATGGAAAGAAGACAAACAGAAAGCGAC |
|  | Reverse | cagcggccgcttaGGTAGTTTCTTTTCGAGAAGTTTTG |
| HA-CDK12 CT | Forward | cggtcgaccATGACCTCAGGGACAAGTACTGAGCCT |
|  | Reverse | cagcggccgcttaGTAAGGAACTCCTCTCCCTCTTCC |
| HA-CDK12 D877N | Forward | AATTTTGGACTTGCTCGGCTCTATAACTCT |
|  | Reverse | TGCTAGTTTGATTTGCCCACTGTTATTCAG |
| HA-CDK12 R858W | Forward | TGGGATATTAAGTGTTCTAACATTTTGCTGAAT |
|  | Reverse | ATGCAGGAAATTCTTTTTGTGACAGTATTCCA |
| HA-CDK12 R882W | Forward | TGGCTCTATAACTCTGAAGAGAGTCGCCCT |
|  | Reverse | AGCAAGTCCAAAATCTGCTAGTTTGATTTGC |
| HA-CDK12 D918G | Forward | GGTGTTTGGAGCTGTGGATGTATTCTTGG |
|  | Reverse | TATGGCTGGTGTGTAACGTTCCTCTCCTA |
| HA-CDK12 R882L | Forward | CTGCTCTATAACTCTGAAGAGAGTCGCCC |
|  | Reverse | AGCAAGTCCAAAATCTGCTAGTTTGATTTGC |
| HA-CDK12 R1008Q | Forward | ATCCTAGTAAGCAGTGCACAGCTGAAC |
|  | Reverse | GTTCAGCTGTGCACTGCTTACTAGGAT |

**Supplementary Table 4. Primers used for RT-qPCR analysis**

| **Genes** | **Primers** | **Sequences** |
| --- | --- | --- |
| *CDK12* | Forward | CCCTGGTGAAGAACAGGACC |
|  | Reverse | GGTGTAACGCTAAGGGGACC |
| *MDM2* | Forward | CTGTGTTCAGTGGCGATTGG |
|  | Reverse | AGGGTCTCTTGTTCCGAAGC |
| *P53* | Forward | CACGAGCTGCCCCCAGG |
|  | Reverse | TCAGTCGACGTCTGAGT |
| *BCL-2* | Forward | CAGAAGGGACTGAATCGGAG |
|  | Reverse | TGGGATGTCAGGTCACTGAA |
| *BCL-XL* | Forward | GACAAGGAGATGCAGGTATTGG |
|  | Reverse | TCCCGTAGAGATCCACAAAAGT |
| *BAX* | Forward | ATGTTTTCTGACGGCAACTTC |
|  | Reverse | ATCAGTTCCGGCACCTTG |
| *BAK* | Forward | CAGATGGATCGCACAGAGAG |
|  | Reverse | TCTGTGTACCACGAATTGGC |
| ERK2 | Forward | TGATCACACAGGGTTCCTGA |
|  | Reverse | TGGAAAGATGGGCCTGTTAG |

**Supplementary Table 5. Information for primary antibodies**

| **Antibodies** | **Source** | **Identifier** | **RRID** |
| --- | --- | --- | --- |
| Anti-Flag | Cell Signaling Technology | Cat#14793 | AB_2572291 |
| Anti-p-RB(S795) | Cell Signaling Technology | Cat#9301 | AB_330013 |
| Anti-RB | Cell Signaling Technology | Cat#9313 | AB_1904119 |
| Anti-p-Pol ll(S2) | Abcam | Cat#ab5095 | AB_304749 |
| Anti-Pol ll | Santa Cruz Biotechnology | Cat# sc-17798 | AB_677355 |
| Anti-ERK2 | Invitrogen | Cat# PA5-29636 | AB_2547111 |
| Anti-p-CDK Substrate | Cell Signaling Technology | Cat#2324 | AB_2244779 |
| Anti-HA | Cell Signaling Technology | Cat#3724 | AB_1549585 |
| Anti-V5 | Cell Signaling Technology | Cat#13202 | AB_2687461 |
| Anti-FOXA1 | Cell Signaling Technology | Cat#53528 | AB_2799438 |
| Anti-CDK12 | Cell Signaling Technology | Cat#11973 | AB_2715688 |
| Anti-Cyclin K | Cell Signaling Technology | Cat#19472 | AB_3674794 |
| Anti-Cleaved Caspase-3 | Cell Signaling Technology | Cat#9661 | AB_2341188 |
| Anti-PARP | Cell Signaling Technology | Cat#9532 | AB_659884 |
| Anti-MDM2 | Cell Signaling Technology | Cat#82504 | AB_3674796 |
| Anti-p53 | Cell Signaling Technology | Cat#2527 | AB_10695803 |
| Anti-BCL-2 | Cell Signaling Technology | Cat#4223 | AB_1903909 |
| Anti-BCL-XL | Cell Signaling Technology | Cat#2764 | AB_2228008 |
| Anti-BAX | Cell Signaling Technology | Cat#2772 | AB_10695870 |
| Anti-BAK | Cell Signaling Technology | Cat#3814 | AB_2290287 |
| Anti-Ki67 | Cell Signaling Technology | Cat#9449 | AB_2797703 |

**Supplementary Table 6. Experimental Models: Cell Lines**

| 22Rv1 | Suggestion: ECACC Cat# 05092802, RRID:CVCL_1045 |
| --- | --- |
| 293T | Suggestion: RRID:CVCL_0063 |
| C4-2 | Suggestion: RRID:CVCL_4782 |
